# *Curcumae radix* Reduces Endoplasmic Reticulum Stress in Mice with Chronic Neuroinflammation

**DOI:** 10.3390/biomedicines11082107

**Published:** 2023-07-26

**Authors:** Seong-Lae Jo, Hyun Yang, Hye Won Lee, Eui-Ju Hong

**Affiliations:** 1 College of Veterinary Medicine, Chungnam National University, Daejeon 34134, Republic of Korea; jsr7093@o.cnu.ac.kr; 2 KM Convergence Research Division, Korea Institute of Oriental Medicine, Daejeon 34054, Republic of Korea; hyunyang@kiom.re.kr

**Keywords:** endoplasmic reticulum (ER) stress, *Curcumae radix*, curcumin, neuroinflammation, chronic inflammation, neurodegenerative diseases

## Abstract

Endoplasmic reticulum (ER) stress is a condition in which the ER protein-folding machinery is impaired, leading to the accumulation of improperly folded proteins and triggering an unfolded-protein response. Excessive ER stress causes cell death and contributes to the development of chronic diseases. Interestingly, there is a bidirectional relationship between ER stress and the nuclear factor-kappa B (NF-κB) pathway. Curcumin, a natural polyphenolic compound found in *Curcumae radix*, exerts its neuroprotective effects by regulating ER stress and inflammation. Therefore, investigating the potential protective and regulatory effects of curcumin on ER stress, inflammation, and neurodegeneration under chronic neuroinflammatory conditions is of great interest. Mice were pretreated with *Curcumae radix* extract (CRE) for 19 days and then treated with CRE plus lipopolysaccharide for 1 week. We monitored pro-inflammatory cytokine levels in the serum and ER stress-, inflammation-, and neurodegeneration-related markers in the mouse cerebrum and hippocampus using Western blotting and qRT-PCR. CRE reduced Interleukin-1 beta levels in the blood and brain of mice with lipopolysaccharide-induced chronic inflammation. CRE also suppressed the expression of markers related to the ER stress and NF-κB signaling pathways. The expression of neurodegeneration-related markers was reduced in the mouse cerebrum and hippocampus. CRE exerts neuroprotective effects under chronic inflammatory conditions via multifaceted anti-inflammatory and ER stress-pathway regulatory mechanisms.

## 1. Introduction

Cellular calcium control, glucose deficiency, and unfolded-protein accumulation cause endoplasmic reticulum (ER) stress [1,2,3]. If the ER stress response is sustained, it can ultimately lead to cell death and contribute to the development of chronic diseases [4,5]. ER stress contributes to the development and progression of neurodegenerative diseases [6,7,8]. Although the etiologies of neurodegenerative diseases remain unclear, studies have shown that ER stress is a common feature of many neurodegenerative disorders and can contribute to the underlying pathology [8]. In Alzheimer’s and Parkinson’s diseases, the accumulation of misfolded proteins, such as amyloid-beta (Aβ) and alpha-synuclein, can induce ER stress and activate the unfolded-protein response (UPR) via three independent ER stress pathways: inositol-requiring enzyme 1 (IRE1), activating transcription factor 6 (ATF-6), and protein kinase R (PKR)-like endoplasmic reticulum kinase (PERK). UPR activation can lead to the generation of pro-apoptotic signaling molecules, which can trigger neuronal death. In addition, ER stress can impair protein-quality management mechanisms and lead to the accumulation of toxic protein aggregates, further exacerbating ER stress and cellular dysfunction. Accumulating evidence suggests that ER stress and UPR dysregulation are the main contributors to the development of neurodegenerative diseases [6,7,8].

ER stress is associated with the regulation of inflammatory responses and plays a role in the development and progression of chronic inflammation [9,10]. Recent studies have shown that ER stress can activate the nuclear factor-kappa B (NF-κB) pathway, which promotes the production of inflammatory cytokines and other mediators [7,11,12]. NF-κB is a protein complex that plays a critical role in regulating the immune response, inflammation, and cell survival [13,14]. ER stress induces IRE1 activation, which recruits TNF Receptor Associated Factor (TRAF) 2 and activates IκB kinase (IKK) complex, ultimately leading to NF-κB activation [15,16,17,18]. Additionally, the PERK pathway can activate NF-κB by inducing eukaryotic Initiation Factor 2 alpha (eIF2α) phosphorylation [18], which induces the production of pro-inflammatory cytokines and chemokines [19]. Furthermore, NF-κB can regulate the UPR by inducing the expression of UPR target genes, such as glucose-regulated protein (GRP)78 and C/EBP homologous protein (CHOP), suggesting a bidirectional relationship between the NF-κB pathway and ER stress [11,18]. They interact with each other and create a feedback loop, where ER stress activates the NF-κB pathway and NF-κB regulates the UPR. In neurodegenerative diseases, including Alzheimer’s and Parkinson’s diseases, NF-κB activation may contribute to neuronal death and degeneration by promoting oxidative stress and inflammation [20,21]. Understanding the relationship between ER stress, NF-κB, and neurodegenerative diseases may provide insights for the development of novel therapeutic strategies for these diseases.

Several studies have demonstrated that certain plant-derived compounds alleviate ER stress and inflammation in neurodegenerative diseases [22,23]. Curcumin, a polyphenolic compound found in the plant *Curcuma longa* L. (*C. longa* L.) [24], has been extensively studied for its role in regulating ER stress and for its anti-inflammatory, anti-cancer, and neuroprotective effects, which include antioxidant, immunomodulatory, anti-angiogenic, hepatoprotective, anti-atherosclerotic, and anti-diabetic effects [25,26,27,28,29,30,31,32,33]. Furthermore, in our previous study, we observed a protective effect of Curcumin Radix, a type of *C. longa* L., against neurodegenerative diseases [34].

Lipopolysaccharides (LPS), consisting of lipids and polysaccharides, a bacterial toxin known as an inflammatory agent, have been widely used to model neuroinflammatory-tory states [35]. LPS-induced neuroinflammation has been shown to activate ER stress and subsequent neuronal injury [36].

Curcumin found in the turmeric rhizome (*C. longa*) has been shown to exert neuroprotective effects by modulating ER stress and inflammation [37,38]. It can inhibit the UPR and reduce the expression of inflammatory cytokines and chemokines [37,39]. However, the efficacy of curcumin in inhibiting ER stress activation and reducing neuroinflammation and neurodegeneration in chronic neuroinflammatory states induced through LPS has not been extensively studied. Based on this evidence, a chronic neuroinflammatory state was modeled using LPS. In this study, we aimed to investigate the efficacy of curcumin in inhibiting ER stress activation and reducing inflammation and neurodegeneration under chronic neuroinflammatory conditions. In particular, we assessed whether curcumin could reduce ER stress and protect neurons.

## 2. Materials and Methods

### 2.1. Curcumae radix Extract Preparation

*Curcumae radix* was obtained from Beneherb Agricultural Co., Ltd. (Jeju Island, Republic of Korea). Sonication was performed twice for 120 min using 70% (*v*/*v*) ethanol to extract the dried powder. Subsequently, the 70% ethanol solution underwent filtration and was concentrated via vacuum rotary evaporation at 40 °C. Finally, the concentrated solution was freeze-dried to yield the *Curcumae radix* powder. A previous study [34] presented a quantitative analysis of the compounds found in *Curcumae radix*.

### 2.2. Animals and Neuroinflammatory Modeling

Six-week-old male Crl:CD-1 (ICR) mice were obtained from Orient Bio Inc. (Seongnam, Republic of Korea). The laboratory mice utilized in this research were raised in a pathogen-free facility at Chungnam National University. They were provided with a standard diet (5L79, Orient Bio Inc.) and had access to water ad libitum. All animal experiments were conducted following the guidelines of the Animal Care Committee of Chungnam Facility. We divided 25 mice into five groups, with each group consisting of five mice: control, lipopolysaccharide, lipopolysaccharide + CRE 10 mg/kg (lipopolysaccharide + CRE 10), lipopolysaccharide + CRE 50 mg/kg (lipopolysaccharide + CRE 50), and lipopolysaccharide + CRE 100 mg/kg (lipopolysaccharide + CRE 100). The mice were acclimated for two weeks before the start of the experiment. For 25 d, the mice were orally administered the CRE extract, and the control group was treated with the same volume of distilled water as the CRE extract. On the 19th d, we treated the mice with lipopolysaccharide (Escherichia coli O111:B4, Sigma-Aldrich, Saint Louis, MO, USA) at a dose of 750 μg/kg/bodyweight for a week. Eight hours before euthanasia, the mice were treated with lipopolysaccharide at a 1.5 mg/kg/body weight dose.

### 2.3. Western Blotting

All protein was extracted from the brain using a protein lysis buffer (78510, Thermo Fisher Scientific, Seoul, Republic of Korea) and quantified using the Bradford assay with PRO-Measure solution (#21011, Intron, Gyeonggi, Republic of Korea). Samples were electrophoresed on 10% polyacrylamide gels and transferred onto PVDF membranes. Following a 1 h blocking step with 3% bovine serum albumin (9048-46-8, LPS solution, Daejeon, Republic of Korea), the membranes were washed with TBS-T buffer (04870517TBST4021, LPS solution) and incubated overnight at 4 °C with primary antibodies (diluted 1:2000). The membranes were washed again with TBS-T and incubated with secondary antibodies (diluted 1:12,000) for 90 min at room temperature. Following TBS-T washes, the membranes were treated with ECL solution (XLS025-0000, Cyanogen, Bologna, Italy); the results were visualized using ChemiDoc (Fusion Solo, VilberLourmat, Collégien, France). Information regarding the primary and secondary antibodies is listed in Table 1.

### 2.4. Total RNA Extraction and Real-Time Quantitative PCR

Total RNA was extracted from the cerebrum and hippocampus using TRIzol reagents (15596-026, Life Technologies, Carlsbad, CA, USA). cDNA synthesis was performed using a reverse transcription kit (SG-cDNAS100; Smartgene, Daejeon, Republic of Korea). Quantitative PCR was carried out using SYBR Green (SG-SYBR-500, SmartGene, Ecublens, Switzerland) on a Stratagene Mx3000P instrument (Agilent Technologies, Santa Clara, CA, USA). The thermal protocol comprised three steps. In the first step, the temperature was maintained at 95 °C for 3 min. The second step involved 40 cycles, with each cycle comprising a denaturation step at 95 °C for 15 s, an annealing step at 60 °C for 15 s, and an extension step at 72 °C for 30 s. Finally, the third segment involved a melt curve analysis, involving a denaturation step at 95 °C for 10 s, an annealing step at 65 °C for 5 s, and a final extension step at 95 °C for 50 s. Transcript levels were determined by analyzing the cycle threshold and monitoring the amplification curve, with *Rplp0* utilized as the internal control. The primer sequences can be found in Table 2.

### 2.5. Serum IL-1β Level

The serum levels of IL-1β (RK00006, Abclonal, Woburn, MA, USA) were measured using ELISA kits according to the manufacturer’s protocol.

### 2.6. Statistical Analysis

Data are indicated as an average ± standard deviation (SD). The differences between means were obtained through a one-way analysis of variance (ANOVA); the Tukey post hoc analysis was then performed using GraphPad Prism Version 8 Software (GraphPad Inc., San Diego, CA, USA).

## 3. Results

### 3.1. CRE Reduces the Neuroinflammatory State Induced by Lipopolysaccharide

**Figure 1:**
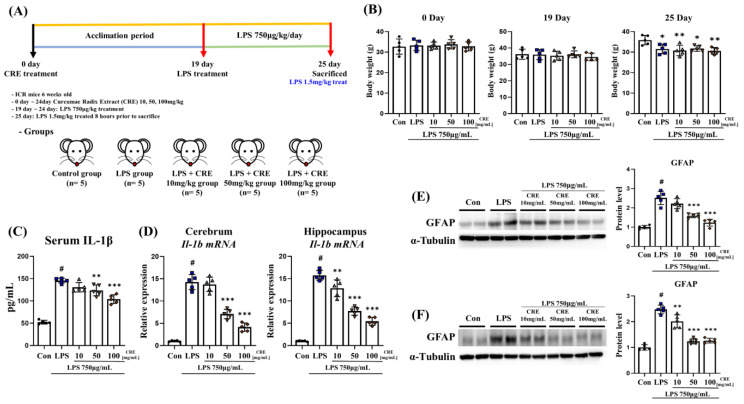
CRE reduces the neuroinflammatory state induced by lipopolysaccharide. (**A**) The experiment outlines the timetable of the animal experiments. The mice were treated with CRE extract orally for 25 d, and the control group was administered distilled water of the same volume as the CRE extract. lipopolysaccharide treatment was given on day 19 at 750 μg/kg/body weight for a week. The mice were sacrificed before 8 h lipopolysaccharide treatment at 1.5 mg/kg body weight, and their organs were collected. (**B**) The changes in mouse body weight on days 0, 19, and 25. The statistical analysis was performed using one-way analysis of variance (ANOVA) and Tukey post hoc analysis. The results are presented as means ± SD, with * *p* < 0.05 and ** *p* < 0.01 indicating significance compared to the lipopolysaccharide group. (**C**) Serum IL-1β levels were measured, with the lipopolysaccharide group showing a significant increase compared to the control group and the lipopolysaccharide + CRE 50 mg/kg and lipopolysaccharide + CRE 100 mg/kg groups showing a significant decrease compared to the lipopolysaccharide group. (**D**) *Il-1b* mRNA levels were measured in the cerebrum and hippocampus of each group of male mice. (**E**) GFAP were evaluated in the cerebrum of each group of male mice by Western blot analysis. α-Tubulin was used as an internal control. (**F**) GFAP were evaluated in the hippocampus of each group of male mice. α-Tubulin was used for internal control. The statistical analysis was performed using one-way ANOVA and Tukey post hoc analysis. The values represent means ± SD # *p* < 0.001 indicating significance compared to the control group and ** *p* < 0.01, *** *p* < 0.001 indicating significance compared to the lipopolysaccharide group.

Six-week-old male ICR mice were treated with CRE extract for 25 d, followed by exposure to lipopolysaccharide for 7 d. Subsequently, the cerebrum and hippocampus were analyzed from the mouse brain tissue (Figure 1A). The body weights of the mice were monitored; no significant differences were observed between the groups on days 0 and 19 (Figure 1B). However, on day 25, the lipopolysaccharide (*p* < 0.05, 88%), lipopolysaccharide + CRE 10 (*p* < 0.01, 86%), lipopolysaccharide + CRE 50 (*p* < 0.05, 89%), and lipopolysaccharide + CRE 100 groups (*p* < 0.01, 86%) showed decreased body weigh compared to the control group (Figure 1B). There were no differences observed between the groups that received lipopolysaccharide treatment. Interleukin-1 beta (IL-1β) is a major inflammatory cytokine in the brain that regulates various physiological and pathological processes and plays a decisive role in the development and progression of neuroinflammatory diseases [40,41]. We measured IL-1β levels in serum and mRNA to investigate whether nerve inflammation was induced through lipopolysaccharide and whether CRE regulates IL-1β in a chronic inflammatory state induced by lipopolysaccharide. The results showed a significant increase (*p* < 0.001, 2.78-fold) in the serum IL-1β levels in the lipopolysaccharide group compared to those in the control group (Figure 1C). The groups treated with lipopolysaccharide + CRE 50 group (*p* < 0.01, 85%) and lipopolysaccharide + CRE 100 group (*p* < 0.001, 71%) exhibited a significant reduction in serum IL-1β levels compared to the lipopolysaccharide group (Figure 1C). *Il-1b* mRNA levels were measured in the cerebrum and hippocampus. In the cerebrum, a significant increase (*p* < 0.001, 14.2-fold) in *Il-1b* mRNA levels was observed in the lipopolysaccharide group compared to the control group. No significant differences were observed between the lipopolysaccharide + 10 and lipopolysaccharide groups. However, the lipopolysaccharide + CRE 50 group (50%) and lipopolysaccharide + CRE 100 group (29%) showed a significant decrease (*p* < 0.001) in *Il-1b* mRNA levels compared to the lipopolysaccharide group (Figure 1D). In the hippocampus, a significant increase (*p* < 0.001, 15.7-fold) in *Il-1b* mRNA levels was observed in the lipopolysaccharide group compared to the control group. The lipopolysaccharide + CRE 10 group (*p* < 0.01, 81%), lipopolysaccharide + CRE 50 group (*p* < 0.001, 49%), and lipopolysaccharide + CRE 100 group (*p* < 0.001, 35%) showed a significant decrease *in Il-1b* mRNA levels compared to the lipopolysaccharide group (Figure 1D). We also measured the protein levels because glial fibrillary acidic protein (GFAP) is involved in astrocyte activation and can be used as a marker of neuroinflammation [42]. In the cerebrum, a significant increase (*p* < 0.001, 2.51-fold) in GFAP protein levels was observed in the lipopolysaccharide group compared to the control group. No significant differences were observed between the lipopolysaccharide + CRE 10 and lipopolysaccharide groups. However, the lipopolysaccharide + CRE 50 (63%) and lipopolysaccharide + CRE 100 (49%) groups showed a significant decrease (*p* < 0.001) in GFAP protein levels compared to the lipopolysaccharide group (Figure 1E). In the hippocampus, a significant increase (*p* < 0.001, 2.5-fold) in GFAP protein levels was observed in the lipopolysaccharide group compared to the control group. The lipopolysaccharide + CRE 10 (*p* < 0.01, 81%), lipopolysaccharide + CRE 50 (*p* < 0.001, 50%), and lipopolysaccharide + CRE 100 groups (*p* < 0.001, 51%) showed a significant decrease in GFAP protein levels compared to the lipopolysaccharide group (Figure 1F).

### 3.2. CRE Decreases Lipopolysaccharide-Induced ER Stress Activation in the Mouse Cerebrum

Previous results have shown that lipopolysaccharide induces IL-1β. Additionally, CRE reduced the use of the secretion of IL-1β. It can promote the activation of UPR and ER stress responses, resulting in a vicious cycle of inflammation and ER stress [43]. We investigated whether ER stress increased during neuroinflammation or whether CRE decreased ER stress.

**Figure 2:**
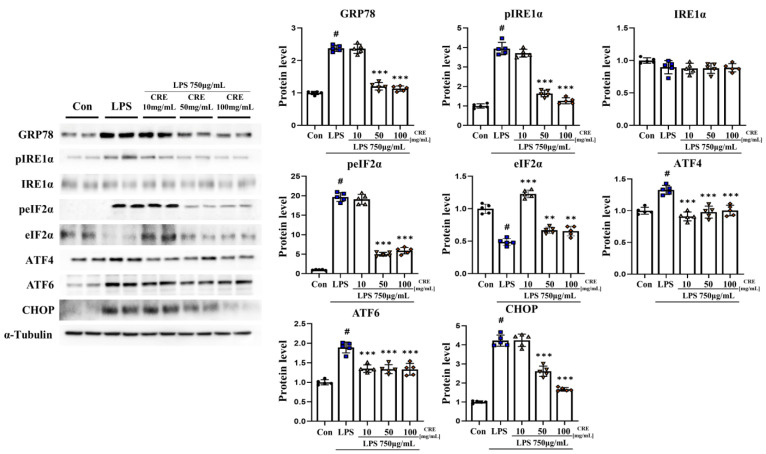
CRE decreases ER stress-related protein markers in the cerebrum. The cerebrum of male mice from each group was analyzed for ER stress-related gene expression using Western blot analysis and quantification, with Alpha-Tubulin as the internal control. The statistical analysis was performed using one-way ANOVA and Tukey post hoc analysis. The values represent means ± SD # *p* < 0.001 indicating significance compared to the control group and ** *p* < 0.01, *** *p* < 0.001 indicating significance compared to the lipopolysaccharide group.

The lipopolysaccharide group exhibited a significant increase (*p* < 0.01, 2.38-fold) in GRP78 protein levels in the cerebrum compared to the control group. However, the groups that received lipopolysaccharide + CRE 50 (51%) and lipopolysaccharide + CRE 100 (48%) showed a significant reduction (*p* < 0.001) in GRP78 protein levels compared to the lipopolysaccharide group (Figure 2). In comparison to the control group, the lipopolysaccharide group displayed a significant increase (3.95-fold, *p* < 0.001) in the phospho-IRE1α (pIRE1α) protein level. The lipopolysaccharide + CRE 50 (42%) and lipopolysaccharide + CRE 100 (32%) groups demonstrated a significant decrease (*p* < 0.001) in the pIRE1α protein level compared to the lipopolysaccharide group (Figure 2). IRE1α protein levels were not significantly different between groups (Figure 2). The lipopolysaccharide group showed a significant increase (19.7-fold, *p* < 0.001) in the phospho-eIF2α (peIF2α) protein level compared to the control group. However, the lipopolysaccharide + CRE 50 group (25%) and lipopolysaccharide + CRE 100 group (30%) groups showed a significant decrease (*p* < 0.001) in the peIF2α protein level compared to the lipopolysaccharide group (Figure 2). The eIF2α protein level exhibited a significant decrease (*p* < 0.05, 49%) in the lipopolysaccharide group compared to the control group. However, The lipopolysaccharide + CRE treatment groups (10 mg/kg, 50 mg/kg, and 100 mg/kg) showed a significant decrease in the eIF2α protein level (2.52-fold, 1.37-fold, and 1.34-fold, respectively, *p* < 0.001) compared to the lipopolysaccharide group (Figure 2). The lipopolysaccharide group showed a significant increase (1.33-fold) in ATF4 protein levels compared to the control group (*p* < 0.001). In the lipopolysaccharide + CRE treatment groups (10, 50, and 100 mg/kg), there was a dose-dependent reduction in ATF4 protein levels (69%, 74%, and 76%, respectively; *p* < 0.001) compared to the lipopolysaccharide group (Figure 2). The lipopolysaccharide group showed a significant increase (*p* < 0.001, 1.89-fold) in ATF6 protein levels compared to the control group. In the lipopolysaccharide + CRE treatment groups (10, 50, and 100 mg/kg), there was a dose-dependent reduction in ATF6 protein levels (72%, 71%, and 71%, respectively; *p* < 0.001) compared to the lipopolysaccharide group (Figure 2). In the lipopolysaccharide group, CHOP protein levels were significantly increased (*p* < 0.001, 4.22-fold) compared to those in the control group. However, the lipopolysaccharide + CRE 50 (62%) and lipopolysaccharide + CRE 100 groups (40%) showed a significant decrease (*p* < 0.001) in CHOP protein levels compared to the lipopolysaccharide group (Figure 2).

### 3.3. CRE Decreases Lipopolysaccharide-Induced ER Stress Activation in the Mouse Hippocampus

**Figure 3:**
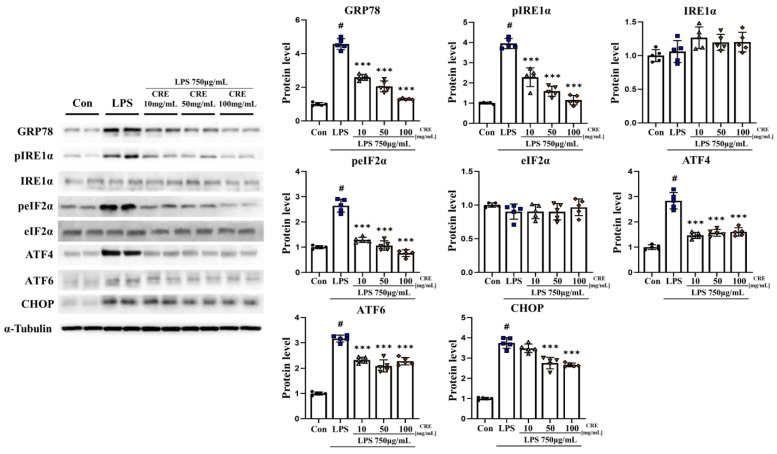
CRE decreases ER stress-related protein markers in the hippocampus. The hippocampus of male mice from each group was analyzed for ER stress-related gene expression using Western blot analysis and quantification, with Alpha-Tubulin as the internal control. The statistical analysis was performed using one-way ANOVA and Tukey post hoc analysis. The values represent means ± SD # *p* < 0.001 indicating significance compared to the control group and *** *p* < 0.001 indicating significance compared to the lipopolysaccharide group.

The GRP78 protein level in the hippocampus increased significantly (*p* < 0.001, 4.57-fold) in the lipopolysaccharide group compared to that in the control group. The lipopolysaccharide + CRE 10 (57%), lipopolysaccharide + CRE 50 (45%), and lipopolysaccharide + CRE 100 groups (28%) showed a dose-dependent decrease (*p* < 0.001) compared to the lipopolysaccharide group (Figure 3). The pIREα protein level increased significantly (*p* < 0.001, 3.95-fold) in the lipopolysaccharide group compared to that in the control group. The lipopolysaccharide + CRE 10 (58%), lipopolysaccharide + CRE 50 (40%), and lipopolysaccharide + CRE 100 groups (29%) showed a dose-dependent decrease (*p* < 0.001) compared to the lipopolysaccharide group (Figure 3). IREα protein levels did not differ significantly between groups (Figure 3). The peIF2α protein level increased significantly (*p* < 0.001, 2.64-fold) in the lipopolysaccharide group compared to that in the control group. In the lipopolysaccharide + CRE treatment groups (10 mg/kg, 50 mg/kg, and 100 mg/kg), there was a dose-dependent reduction in peIF2α protein levels (49%, 40%, and 29%, respectively, *p* < 0.001) compared to the lipopolysaccharide group (Figure 2).

The eIF2α protein levels did not differ significantly among all groups (Figure 3). The ATF4 protein level increased significantly (2.84-fold) in the lipopolysaccharide group compared to that in the control group (*p* < 0.001). In the lipopolysaccharide + CRE treatment groups (10, 50, and 100 mg/kg), there was a dose-dependent reduction in ATF4 protein levels (52%, 55%, and 57%, respectively; *p* < 0.001) compared to the lipopolysaccharide group (Figure 3). The ATF6 protein level increased significantly (*p* < 0.001, 3.17-fold) in the lipopolysaccharide group compared to the control group. In the lipopolysaccharide + CRE treatment groups (10, 50, and 100 mg/kg), there was a dose-dependent reduction in ATF4 protein levels (73%, 66%, and 72%, respectively; *p* < 0.001) compared to the lipopolysaccharide group. CHOP protein levels increased significantly (3.74-fold) in the lipopolysaccharide group compared with those in the control group (*p* < 0.001). The lipopolysaccharide + CRE 50 (74%) and lipopolysaccharide + CRE 100 groups (71%) showed a significant decrease (*p* < 0.001) in CHOP protein levels compared to the lipopolysaccharide group (Figure 3).

### 3.4. CRE Decreases Lipopolysaccharide-Induced NF-κB and Apoptosis Activation in Mouse Cerebrum

**Figure 4:**
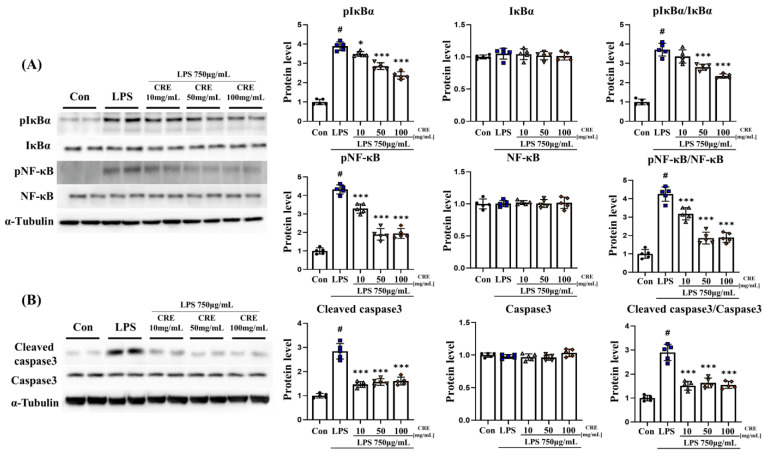
CRE decreases the NF-κB pathway and apoptosis-related protein levels in the cerebrum. (**A**) The levels of NF-κB pathway-related genes in the cerebrum of male mice from each group were analyzed using Western blot and quantification methods, with alpha-tubulin as the internal control. (**B**) The levels of apoptosis-related genes in the cerebrum of male mice from each group were analyzed using Western blot and quantification methods, with Alpha-Tubulin as the internal control. The statistical analysis was performed using one-way ANOVA and Tukey post hoc analysis. The values represent means ± SD # *p* < 0.001 indicating significance compared to the control group and * *p* < 0.05, *** *p* < 0.001 indicating significance compared to the lipopolysaccharide group.

The relationship between ER stress and NF-κB is complex and bidirectional, and both processes affect each other [18]. Our results confirmed that IL-1β and ER stress was increased by lipopolysaccharide and decreased by CRE treatment. Accordingly, we hypothesized that the NF-κB pathway is activated. The protein level of phospho-IκBα (pIκBα) in the cerebrum increased significantly (*p* < 0.001, 3.89-fold) in the lipopolysaccharide group compared to the control group. However, in the lipopolysaccharide + CRE treatment groups (10 mg/kg, 50 mg/kg, and 100 mg/kg), there was a dose-dependent reduction of pIκBα protein level (90%, 73%, and 61%, respectively) compared to the lipopolysaccharide group (Figure 4A), with statistical significance observed in the lipopolysaccharide + CRE 50 (*p* < 0.001, 75%) and lipopolysaccharide + CRE 100 (*p* < 0.05, 63%) groups. There was no significant difference in IκBα protein levels among the groups. However, the pIκBα/IκBα ratio increased significantly (*p* < 0.001, 3.71-fold) in the lipopolysaccharide group compared to the control group. However, this ratio decreased dose-dependently in the lipopolysaccharide + CRE treatment groups (50 mg/kg and 100 mg/kg) (75% and 63%, respectively, *p* < 0.001) (Figure 4A). In addition, the protein level of phospho-NF-κB (pNF-κB) in the cerebrum significantly increased (*p* < 0.001, 4.33-fold) in the lipopolysaccharide group compared to the control group. Nonetheless, the lipopolysaccharide + CRE treatment groups (50 mg/kg and 100 mg/kg) showed a dose-dependent decrease in pNF-κB protein level (76%, 44%, and 45%, respectively, *p* < 0.001) (Figure 4A). There was no significant difference in NF-κB protein levels among the groups. However, the pNF-κB/NF-κB ratio increased significantly (*p* < 0.001, 4.25-fold) in the lipopolysaccharide group compared to the control group. The lipopolysaccharide + CRE treatment groups (10 mg/kg, 50 mg/kg, and 100 mg/kg) showed a significant decrease in the pNF-κB/NF-κB ratio (75%, 44%, and 44%, respectively, *p* < 0.001) compared to the lipopolysaccharide group (Figure 4A). Additionally, Not only is NF-κB known to promote apoptosis, but it has also been studied that CHOP, which activates apoptosis, is necessary to activate NF-κB [44,45]. We hypothesized that increased CHOP levels would ultimately lead to increased apoptosis. To assess caspase-3 levels, we analyzed the cerebrum of mice from each group. The cleaved caspase-3 protein level was significantly higher in the lipopolysaccharide group than in the control group (*p* < 0.001, 2.84-fold). However, the lipopolysaccharide + CRE treatment groups (10, 50, and 100 mg/kg) showed a significant decrease in cleaved caspase-3 protein levels (52%, 55%, and 57%, respectively; *p* < 0.001) compared to the lipopolysaccharide group (Figure 4B). Caspase-3 protein did not differ significantly between the groups. Nonetheless, the ratio of cleaved caspase-3 to caspase-3 significantly increased (*p* < 0.001, 2.90-fold) in the lipopolysaccharide group compared to that in the control group. The lipopolysaccharide + CRE treatment groups (10, 50, and 100 mg/kg) showed a significant decrease in the ratio of cleaved caspase-3 to caspase-3 (52%, 55%, and 57%, respectively, *p* < 0.001) compared to the lipopolysaccharide group (Figure 4B).

### 3.5. CRE Decreases Lipopolysaccharide-Induced NF-κB and Apoptosis Activation in the Mouse Hippocampus

**Figure 5:**
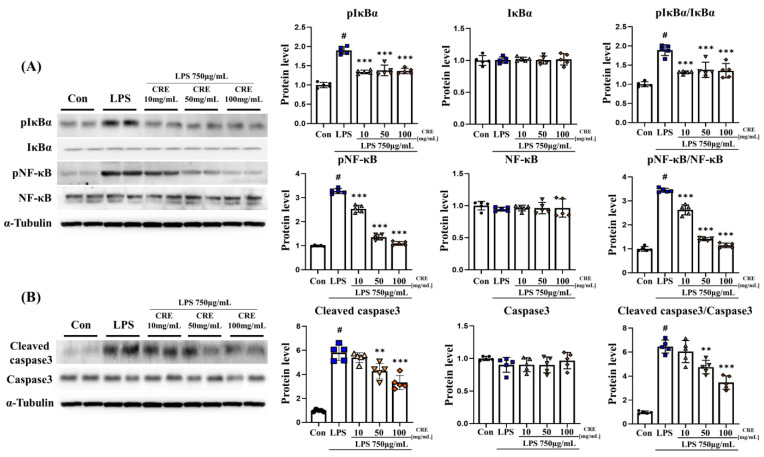
CRE decreases the NF-κB pathway and apoptosis-related protein levels in the hippocampus. (**A**) The levels of NF-κB pathway-related genes in the hippocampus of male mice from each group were analyzed using Western blot and quantification methods, with Alpha-Tubulin as the internal control. (**B**) The levels of apoptosis-related genes in the hippocampus of male mice from each group were analyzed using Western blot and quantification methods, with Alpha-Tubulin as the internal control. The statistical analysis was performed using one-way ANOVA and Tukey post hoc analysis. The values represent means ± SD # *p* < 0.001 indicating significance compared to the control group and ** *p* < 0.01, *** *p* < 0.001 indicating significance compared to the lipopolysaccharide group.

The protein level of pIκBα in the hippocampus increased significantly (*p* < 0.001, 1.89-fold) in the lipopolysaccharide group compared to the control group. The lipopolysaccharide + CRE treatment groups (10 mg/kg, 50 mg/kg, and 100 mg/kg) showed a significant decrease in pIκBα protein level (71%, 73%, and 72%, respectively, *p* < 0.001) compared to the lipopolysaccharide group (Figure 5A). There was no significant difference in IκBα protein levels among the groups. The pIκBα/IκBα ratio increased significantly (*p* < 0.001, 1.89-fold) in the lipopolysaccharide group compared to the control group, The lipopolysaccharide + CRE treatment groups (10 mg/kg, 50 mg/kg, and 100 mg/kg) showed a significant decrease in pIκBα protein level (69%, 73%, and 72%, respectively, *p* < 0.001) compared to the lipopolysaccharide group (Figure 5A). In addition, the protein level of pNF-κB in the cerebrum was significantly increased (*p* < 0.001, 3.28-fold) in the lipopolysaccharide group compared to that in the control group. The lipopolysaccharide + CRE treatment groups (10, 50, and 100 mg/kg) showed a significant dose-dependent decrease in pNF-κB protein levels (78%, 42%, and 34%, respectively; *p* < 0.001) compared to the lipopolysaccharide group (Figure 5A). The NF-κB protein levels did not change significantly among all groups. However, the pNF-κB/NF-κB ratio increased significantly (*p* < 0.001, 3.44-fold) in the lipopolysaccharide group. The lipopolysaccharide + CRE treatment groups (10 mg/kg, 50 mg/kg, and 100 mg/kg) showed a dose-dependent significant decrease in the pNF-κB/NF-κB ratio (76%, 41%, and 33%, respectively, *p* < 0.001) compared to the lipopolysaccharide group (Figure 5A). The cleaved caspase3 protein level increased significantly (5.95-fold) in the lipopolysaccharide group compared to that in the control group (*p* < 0.001). The lipopolysaccharide + CRE 50 (*p* < 0.01, 74%) and lipopolysaccharide + CRE 100 groups (*p* < 0.001, 57%) showed a significant decrease compared with the lipopolysaccharide group (Figure 5B). The caspase-3 protein levels did not differ significantly between the groups. However, the ratio of cleaved caspase-3 to caspase-3 increased significantly (*p* < 0.001, 6.61-fold) in the lipopolysaccharide group compared with that in the control group. Furthermore, the lipopolysaccharide + CRE 50 (*p* < 0.01, 74%) and lipopolysaccharide + CRE 100 (*p* < 0.001, 54%) groups showed a significant decrease compared to the lipopolysaccharide group (Figure 5B).

### 3.6. CRE Weakens Neurodegenerative Diseases Caused by Chronic Neuroinflammation

**Figure 6:**
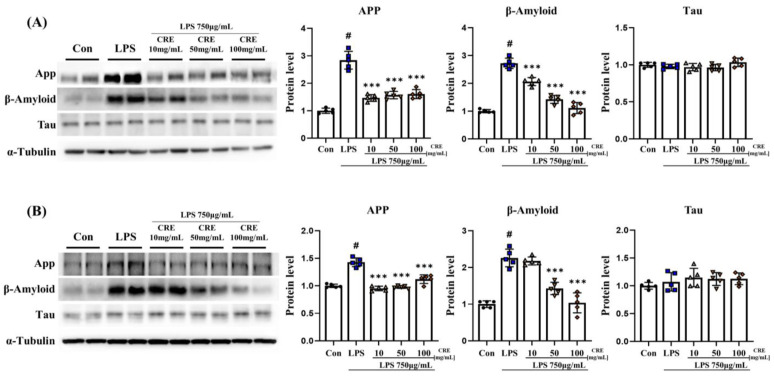
CRE reduces Alzheimer’s disease-related markers in mouse brains. (**A**) The levels of Alzheimer’s disease-related features in the cerebrum of male mice from each group were analyzed using Western blot and quantification methods, with Alpha-Tubulin as the internal control. (**B**) The levels of Alzheimer’s disease-related markers in the hippocampus of male mice from each group were analyzed using Western blot and quantification methods, with Alpha-Tubulin as the internal control. The statistical analysis was performed using one-way ANOVA and Tukey post hoc analysis. The values represent means ± SD # *p* < 0.001 indicating significance compared to the control group and *** *p* < 0.001 indicating significance compared to the lipopolysaccharide group.

Overall, ER stress activation, inflammation, and apoptosis were increased by lipopolysaccharide induction; CRE-suppressed factors were directly involved in neurodegeneration. We measured App, Aβ, and Tau proteins to see changes in neurodegenerative-related markers due to ER stress activation, inflammation, and apoptosis, and the neuroprotective effects of CRE. In the cerebrum, the lipopolysaccharide group showed a significant increase (*p* < 0.001, 2.84-fold) in the App protein level compared to the control group, while the lipopolysaccharide + CRE 10 (52%), lipopolysaccharide + CRE 50 (55%), and lipopolysaccharide + CRE 100 (57%) groups exhibited a noteworthy (*p* < 0.001) decrease in comparison to the lipopolysaccharide group (Figure 6A). Furthermore, the Aβ protein levels in the lipopolysaccharide group displayed a significant increase (*p* < 0.001, 2.72-fold) in contrast to the control group. However, the lipopolysaccharide + CRE 10 (76%), lipopolysaccharide + CRE 50 (52%), and lipopolysaccharide + CRE 100 (41%) groups showed a dose-dependent decrease (*p* < 0.001) compared to the lipopolysaccharide group (Figure 6A). Tau protein levels did not differ significantly between groups.

In the hippocampus, the App protein level in the lipopolysaccharide group was significantly increased (2.26-fold) compared to that in the control group (*p* < 0.001). Nonetheless, the lipopolysaccharide + CRE 10 (67%), lipopolysaccharide + CRE 50 (68%), and lipopolysaccharide + CRE 100 (78%) groups showed a significant (*p* < 0.001) decrease compared to the lipopolysaccharide group (Figure 6B). Similarly, the Aβ protein levels in the lipopolysaccharide group exhibited a significant increase (*p* < 0.001, 2.84-fold) compared to the control group. In comparison, the lipopolysaccharide + CRE 50 (63%) and lipopolysaccharide + CRE 100 (46%) groups showed a significant (*p* < 0.001) decrease compared to the lipopolysaccharide group (Figure 6B). Tau protein levels did not differ significantly between groups.

## 4. Discussion

Neurodegenerative diseases are complex and multifactorial, involving various genetic, environmental, and lifestyle factors. In recent years, interest in the role of ER stress in the pathogenesis of neurodegenerative diseases has increased [6,7,8]. ER stress and chronic inflammation are closely associated with the development of neurodegenerative diseases [6,9,46]. ER stress leads to the production and secretion of inflammatory cytokines and chemokines [47]. Conversely, inflammation can further exacerbate ER stress by activating various signaling pathways that promote incorrect folding and aggregation of proteins [48]. These processes create a vicious cycle and deteriorate each other, ultimately leading to neurological dysfunction and death [49]. A greater understanding of the association between ER stress and neuroinflammation can help identify new treatment approaches for neurodegenerative diseases. Natural products have been extensively studied owing to their potential therapeutic effects on various diseases [50,51]. As a result, numerous natural products that modulate ER stress and inflammation have been identified, making them promising candidates for developing new treatments for neurodegenerative diseases [22,23,52,53]. One of the advantages of using natural products to treat ER stress and inflammation is that they have relatively low toxicity, multiple targets, and exert pleiotropic effects, making them more effective than single-target synthetic drugs [54,55].

Neuroinflammation is a complex process involving the activation of glial cells, such as microglia and astrocytes, in response to various stimuli, including injury, infection, and neurodegeneration [56,57]. Activated neuroglial cells contribute to the development and progression of neuroinflammatory diseases by secreting inflammatory cytokines, including Interleukin-1beta (IL-1β) [37,58]. Our results confirmed that the lipopolysaccharide-treated group ex-habited neuroinflammatory effects, as demonstrated by elevated levels of IL-1β and GFAP. However, neuroinflammation was reduced following CRE treatment, indicating that CRE, containing curcumin, directly regulated the levels of pro-inflammatory cytokines and reduced lipopolysaccharide-induced neuroinflammation.

Interestingly, ER stress can induce the production of inflammatory cytokines, such as IL-1β, by activating the UPR, while IL-1β can induce ER stress by activating inflammation-related signals [47,59,60]. Based on this positive feedback, we anticipated that CRE lowers ER stress by reducing IL-1β levels. ER stress occurs when proteins are unfolded or misfolded in the ER lumen, interfering with the protein-folding process and leading to cell death. IRE1, ATF6, and PERK mediate the three main UPR pathways. GRP78 plays an essential role in maintaining proper ER functionality, while IRE1 is involved in ER homeostasis, PERK inhibits protein synthesis to reduce the ER load, and ATF6 activates protein-folding-related genes in response to ER stress, leading to increased cell death under prolonged ER stress [5,61,62]. Studies have shown that curcumin reduces ER stress by inhibiting the expression of major UPR proteins, such as PERK, IRE1α, and ATF6, and by directly regulating the expression of chaperone proteins, such as GRP78 [25,35]. Our results showed that the expression of ER stress-related genes in mouse cerebrum and hippocampus was lower in the CRE-treated group than in the lipopolysaccharide-treated group. Therefore, we suggest that CRE reduces ER stress by directly regulating the expression of key proteins in the UPR, promoting proper ER function, and preventing ER stress-related diseases.

IL-1β induces ER stress by activating inflammatory signaling pathways [43,63]. Our results confirmed that CRE regulates IL-1β expression. In inflammatory and immune responses, IL-1β-mediated NF-κB activation induces the expression of inflammatory cytokines [13]. Therefore, we hypothesized that CRE, a natural product with anti-inflammatory effects, reduces the activation of the NF-κB pathway. Here, we observed less NF-κB activation in the CRE-treated group. This may be owing to a decrease in IL-1β due to CRE-mediated inhibition of NF-κB activity or a decrease in IL-1β-mediated NF-κB activity [64,65]. Overall, the effect of CRE on chronic neuroinflammation induced by LPS resulted in a reduction in the activation of IL-1β, an inflammatory cytokine that plays a decisive role in the immune response, as well as NF-κB, a transcription factor that regulates the expression of various genes involved in the inflammatory response. Additionally, the treatment of CRE demonstrated the ability to down-regulate GFAP, a neuroinflammatory-related marker, which showed an up-regulation in expression with LPS administration. Therefore, the anti-inflammatory ability of CRE appears to have contributed to the reduction of neuroinflammatory markers such as IL-1β and NF-κB. By inhibiting these markers, CRE may have helped weaken the neuroinflammatory response. Additionally, CRE can be suggested that the potential down-regulation of GFAP expression could further contribute to the mitigation of neuroinflammatory activity.

As NF-κB activation can induce ER stress by increasing the expression of several ER chaperones [11,18], CRE-mediated inhibition of NF-κB can reduce ER stress. Ultimately, ER stress and NF-κB activation are involved in apoptosis, and the expression of apoptosis-related genes can provide insights into this process [45,66,67]. In this study, the cleaved caspase3/caspase3 ratio was lower in the CRE-treated group than in the lipopolysaccharide-treated group, suggesting that CRE may have a protective effect against cell death.

Pro-inflammatory cytokines regulate genes related to Alzheimer’s disease [68,69], and NF-κB is a known risk factor for Alzheimer’s disease-related neurodegeneration [21,70]. NF-κB activation leads to the induction of APP/BACE1, which further causes the accumulation of Aβ [71]. Activated astrocytes produce inflammatory IL-1β and inducible nitric oxide synthetases that produce neurotoxic free radicals [72]. When excessive ER stress produces Aβ and accumulates Aβ peptides in Alzheimer’s disease, the increased Aβ peptides exacerbate ER stress by overloading the ER, further increasing reactive oxygen species production [73,74]. Thus, there appears to be a vicious cycle involving ER stress, Aβ production, oxidative stress, and ultimately, cell apoptosis. In the present study, we observed lower levels of APP and Aβ in the CRE-treated group than in the lipopolysaccharide-treated group. These findings suggest that curcumin-containing CRE exerts a neuroprotective effect via the multifaceted regulation of the anti-inflammatory and ER stress pathways.

To use curcumin as a preventive and therapeutic agent for neurodegeneration, its low bioavailability and limited ability to cross the blood-brain barrier should be considered. [75]. Strategies to enhance its effectiveness include nanotechnology-based delivery systems, liposomal curcumin, and targeted delivery to specific brain regions and cell types [76,77,78]. It is also important to optimize the dose and duration of treatment to minimize potential side effects and to monitor any pro-oxidant effects at high doses or in certain contexts [54,79]. Despite efforts to improve its bioavailability, further research is warranted to explore ways to increase its systemic effectiveness and facilitate its use in clinical settings. Additionally, it has been reported that the regulation of neuroinflammatory processes typical of many natural compounds exerts a specific activity on synaptic efficiency in the framework of neurodegenerative diseases [80]. Therefore, we suggest that it is also necessary to analyze synaptic activity indicators for future studies.

## 5. Conclusions

CRE, a curcumin-containing compound, offers neuroprotection in chronic neuroinflammatory conditions by regulating multiple aspects of ER stress, inflammation, and apoptosis. These findings suggest that CRE has the potential to serve as an effective treatment for chronic neuroinflammatory and neurodegenerative diseases.

## Figures and Tables

**Table 1:** Antibodies.

Primary Antibodies	Type	Lot.	Inc.
GRP78	Rabbit monoclonal	GTX113340	Genetex, Inc. (Irvine, CA, USA)
Phospho-IRE1α	Rabbit monoclonal	GTX63722	Genetex, Inc.
IRE1α	Rabbit monoclonal	ab37073	Abcam, Inc. (Waltham, MA, USA)
Phospho-eIF2α	Rabbit monoclonal	#3597	Cell signaling technology (Danvers, MA, USA)
eIF2α	Rabbit monoclonal	#9722	Cell signaling technology
ATF4	Rabbit monoclonal	#11815	Cell signaling technology
ATF6	Rabbit monoclonal	ab65838	Abcam, Inc.
phospho-IκBα	Rabbit monoclonal	#2697	Cell signaling technology
IκBα	Mouse monoclonal	#4814	Cell signaling technology
phospho-NF-κB	Rabbit monoclonal	#3033	Cell signaling technology
NF-κB	Rabbit monoclonal	#8242	Cell signaling technology
phospho-STAT3	Rabbit monoclonal	AP0070	Company ABclonal, Inc.
STAT3	Mouse monoclonal	A1192	Company ABclonal, Inc.
Cleaved caspase3	Rabbit monoclonal	#9664	Cell signaling technology
Caspase3	Rabbit monoclonal	#9665	Cell signaling technology
GFAP	Rabbit monoclonal	A19058	Company ABclonal, Inc.
Amyloid-beta	Mouse monoclonal	sc-28365	Santa Cruz Biotechnology (Santa Cruz, CA, USA)
Tau	Rabbit monoclonal	A1103	Company ABclonal, Inc.
Secondary Antibodies	Type	Lot.	Inc.
Anti-Mouse IgG	Goat	121507	Jackonimmuno (West Grove, PA, USA)
Anti-Rabbit IgG	Mouse	123213	Jackonimmuno

**Table 2:** Primers.

Gene	Forward Primer (5′-3′)	Reward Primer (5′-3′)	Species
*Il-1b*	GCC CAT CCT CTG TGA CTC AT	AGG CCA CAG GTA TTT TGT CG	Mouse
*Rplp0*	GCA GCA GAT CCG CAT GTC GCT CCG	GAG CTG GCA CAG TGA CCT CAC ACG G	Mouse

## Data Availability

Not applicable.
